# Lipid Droplets in Neurodegenerative Disorders

**DOI:** 10.3389/fnins.2020.00742

**Published:** 2020-07-29

**Authors:** Brandon C. Farmer, Adeline E. Walsh, Jude C. Kluemper, Lance A. Johnson

**Affiliations:** ^1^Department of Physiology, University of Kentucky, Lexington, KY, United States; ^2^Sanders Brown Center on Aging, University of Kentucky, Lexington, KY, United States

**Keywords:** lipid droplet, CNS, astrocytes, microglia, fatty acids, neurodegeneration, brain

## Abstract

Knowledge of lipid droplets (LDs) has evolved from simple depots of lipid storage to dynamic and functionally active organelles involved in a variety of cellular functions. Studies have now informed significant roles for LDs in cellular signaling, metabolic disease, and inflammation. While lipid droplet biology has been well explored in peripheral organs such as the liver and heart, LDs within the brain are relatively understudied. The presence and function of these dynamic organelles in the central nervous system has recently gained attention, especially in the context of neurodegeneration. In this review, we summarize the current understanding of LDs within the brain, with an emphasis on their relevance in neurodegenerative diseases.

## Introduction

Lipid droplets (LDs) are spherical organelles that store intracellular neutral lipid such as triacylglycerols (TAGs) and cholesteryl esters (CEs; Welte, 2015; Cohen, 2018). LDs serve as lipid reservoirs for cells by providing substrates for membrane formation and energy metabolism (Walther and Farese, 2012). Adipose tissue is the most LD-enriched tissue in the body, where fatty acids are stored in times of nutrient excess and then mobilized with increased energy demand (Missaglia et al., 2019). LDs also affect physiological processes in the periphery beyond simple fatty acid storage and supply, such as in inflammation and insulin signaling. For example, LDs in various immune cell types contain a large pool of intracellular arachidonic acid (AA), which provides a reserve of precursors for eicosanoid synthesis (Bozza and Viola, 2010; Saka and Valdivia, 2012; Dichlberger et al., 2016). Enzymes involved in AA processing have been demonstrated to LDs, indicating that these organelles serve as a supply site for inflammation (Bozza et al., 2011). Additionally, LDs have been linked to peripheral metabolic dysfunction such as ectopic lipid accumulation (Puri et al., 2007) and insulin resistance (Gemmink et al., 2017). Overexpression of LD-associated proteins such as Cidea increase fat accumulation in mice, and human expression of LD proteins in adipose correlates with clinical insulin resistance (Puri et al., 2008). These studies therefore suggest a role for LDs in obesity-driven metabolic dysregulation.

Lipid droplets may also affect cellular physiology and function in the central nervous system (CNS). The brain is the second most lipid-rich organ (Hamilton et al., 2007), storing 20% of the body’s total cholesterol (Zhang and Liu, 2015). Alteration in the lipid composition of CNS cells has been shown to affect cell function and normal neural activity (Puchkov and Haucke, 2013; Bruce et al., 2017). Notably, neurodegenerative diseases, including Alzheimer’s disease (AD), and Parkinson’s disease (PD), share lipid dysregulation as a metabolic feature in disease pathology. In this review, we discuss evolving knowledge and recent advances in understanding the contribution of LDs to pathogenesis of neurodegenerative diseases. Growing knowledge of LDs in the CNS is important to the advancement of the field, as these dynamic organelles may reveal common mechanisms and potential therapeutic targets to neurodegenerative disease.

## LDS Structure, Composition, and Biogenesis

**FIGURE 1:**
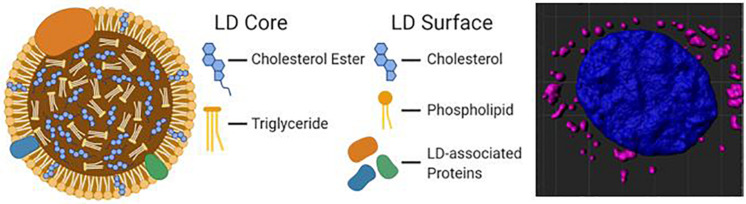
A diagram of the molecular structure of a standard lipid droplet (LD) along with a software reconstruction of a microscopic image of astrocytes *in vitro* containing lipid droplets (highlighted in pink; LipidSpot) surrounding a nucleus (in blue; DAPI).

Hydrophobic molecules such as TAGs, CEs, and retinyl esters constitute the core of a lipid droplet (Meyers et al., 2017), while the outer surface is formed by an amphipathic lipid monolayer embedded with LD-associated proteins (The general structure of an LD is illustrated in Figure 1). Additionally, proteins can reside in the LD core depending on the cell type. This unique monolayer distinguishes LDs from organelles of similar size, such as lysosomes and endosomes, as the latter exhibit a lipid bilayer. Of the many LD-associated proteins, members of the perilipin family have been well described for their essential roles in LD metabolic regulation (Sztalryd and Brasaemle, 2017). The exterior protein components of LDs allow for a variety of unique interactions that may explain the myriad of cellular roles accomplished via LDs in energy homeostasis, cellular communication, and disease.

Lipid droplets arise from the endoplasmic reticulum (ER) by budding off the cytoplasmic leaflet of the ER membrane (Walther et al., 2017). They are comprised of acyl-glycerols that are synthesized through the action of diacylglycerol transferases (DGATs), which convert acyl-CoA-bound fatty acids, and diacylglycerols (DAGs) into the TAGs that fill the LD core (Harris et al., 2011). Cholesterol acyltransferases synthesize CEs which are also incorporated into the core of nascent LDs (Zhu et al., 2018). Once separated from the ER membrane, LDs may continue to grow via LD fusion and further TAG incorporation. Fusion of LDs with the aid of members of Cell death-inducing DFF45-like effector (CIDE) family proteins (Gao et al., 2017) coalesce smaller LDs into larger LDs. Re-localization of TAG synthesis enzymes like DGAT2 and GPAT4 from the ER to the LD surface allows direct synthesis of TAGs from cellular lipid sources (Wilfling et al., 2013), such as fatty acids derived from autophagic phospholipid breakdown (Nguyen et al., 2017). The incorporation of cellular debris into LDs is commonly seen during periods of stress and starvation and are thought to protect the cells from lipotoxicity (Rambold et al., 2015). LDs exhibit a variety of protein and lipid signatures, and these various compositions can help determine LD localization and utilization. For example, perilipin 2 (PLIN2) has relatively low control over lipolysis, so LDs that contain PLIN2 may be more easily broken down. Conversely, PLIN1 and PLIN5 actively promote lipolysis when activated. PLIN1 acts through its release of CGI-58, a co-activator of adipose triglyceride lipase (ATGL; Lass et al., 2006), while PLIN5 binds directly to ATGL to promote lipolysis (Wang et al., 2011). Therefore, LDs with varying PLIN proteins will behave differently across various tissues and environmental conditions (Sztalryd and Brasaemle, 2017).

Lipid homeostasis is necessary for maintaining neuronal function and synaptic plasticity (Montesinos et al., 2020), and dynamic interaction between perilipins and lipases on the LD surface regulate cellular lipid storage, breakdown, and metabolism (Olzmann and Carvalho, 2019). In most cells, the bulk of LD breakdown is accomplished through ATGL, another LD outer layer protein (Etschmaier et al., 2011). Lipophagy is also a recognized LD breakdown process, in which an LD is taken up into an autophagosome and subsequently fuses with a lysosome to breakdown LD contents mainly through lipid acid lipases (Cingolani and Czaja, 2016). During times of metabolic stress, lipases cleave triglycerides into FAs, which are then processed in the mitochondria to liberate the energy stored in droplets via beta-oxidation into acetyl-CoA and subsequent TCA cycle activity and oxidative phosphorylation (Zechner et al., 2012). LDs have been shown to provide energy substrates (Cabodevilla et al., 2013; Farmer et al., 2019), lipid signaling molecules (Arrese et al., 2014), and membrane infrastructure materials (Zehmer et al., 2009) for various cell types.

## LDS Formation in Cells of the Brain

**FIGURE 2:**
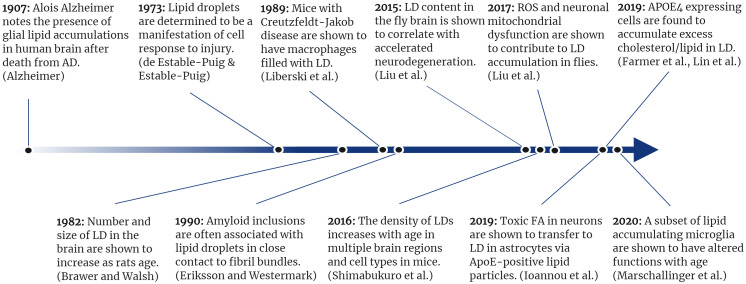
A timeline of selected lipid droplet discoveries as they relate to neurodegenerative disease.

Essentially all brain cell types have been shown to form LDs (Table 1 and Figure 2). A recent study claimed that the majority of brain LDs (stained with the fluorescent neutral lipid probe BODIPY) were co-localized with ionized calcium binding adaptor molecule 1 (Iba1), a microglia/macrophage-specific protein. This finding implicates microglia as a main harbor of LDs (Marschallinger et al., 2020). These lipid-associated microglia had a unique transcriptomic signature compared to non-LD-laden microglia, suggesting that LDs in microglia are either a cause or result of substantial transcriptional modulations. However, subsequent co-localization using other brain cell-specific markers was not reported in this study, leaving the door open for other cell types to be involved. For example, ependymal cells that line the cerebral ventricular system have been shown to accumulate LDs, along with Glial fibrillary acidic protein (GFAP) positive cells that are closely associated in the ependymal niche (GFAP is a common marker for astrocytes and ependymal cells; Hamilton and Fernandes, 2018). Lesions to the CNS have also induced LDs in neurons and astrocytes (Ioannou et al., 2019), and glial cells have been shown to form LDs from phagocytosed myelin fragments (Lee et al., 1990). Finally, a thorough immunofluorescence study examining cell types that harbor PLIN positive droplets found Iba1+, GFAP+, NeuN+ (a neuron specific antibody), and S100β+ (a calcium binding protein that is localized in astrocytes) to colocalize with droplets (Shimabukuro et al., 2016). Together, these studies and others demonstrate that multiple cell types in the brain are capable of forming LDs.

**TABLE 1:** Lipid droplet-related literature pertaining to the brain.

Model organism	Area of interest	Cell type	Author and year of publication	Summary of findings
Human	Frontal cortex	Neuron	Paula-Barbosa et al., 1980	LD-like vesicles visible in cortical dendrites that had abundant degeneration.
	Frontal lobe	Astrocyte	Miyazu et al., 1991	Sudanophilic LDs observed in the thalamus of a patient with Nasu-Hakola disease.
	Medial temporal	Various	Derk et al., 2018	DIAPH1 colocalize with LD accumulation in myeloid cells.
	Choroid plexus	Adrenal Cortical	Eriksson and Westermark, 1990	Amyloid inclusions associated with LDs in close contact to fibril bundles.
	Whole brain	Neuron	Hulette et al., 1992	Brain biopsy found ballooned neurons filled with oligolamellar cytosomes and LDs.
			Ozsvar et al., 2018	Demyelination debris contribute to LD formation; volume highest in corpus callosum
Rat	Cerebral cortex	Neuron	Smialek et al., 1997	Injection of squalene led to LD accumulation in myelin sheaths of neurons.
	Hippo-campus	Neuron	Ahdab-Barmada et al., 1986	Excess oxygen caused neuronal necrosis. Neurons accumulate electron dense LD.
			Cole et al., 2002	The protein α-synuclein was less effective at regulating TAG turnover and showed variable distribution on LDs.
	Median eminence	Tanycyte	Brawer and Walsh, 1982	The number and size of LDs increased with age.
	Olfactory bulbs	Neurons	de Estable-Puig and Estable-Puig, 1973	LDs are manifestations of cell response to injury.
	Perineurium	Perineurial glia	Benstead et al., 1989	LD formation was found to be an early reactive change to ischemia in perineurial, endothelial, and Schwann cells.
	Pineal gland	Pinealocyte	Johnson, 1980	Removal of the hypophysis led to significant loss of LDs in the pineal gland.
	Pituitary gland	Folliculostellate	Stokreef et al., 1986	Folliculostellate cells became packed with LDs after estrogen withdrawal.
		Neuron	Gajkowska and Zareba-Kowalska, 1989	Supraopticus and paraventricularis neurons show increased LDs post-ischemia.
	Striatum	Neuron	Marasigan et al., 1986	LDs formed in neurons of rats injected with kainic acid.
	Whole brain	Glia	Kamada et al., 2003	Macrophages and astrocytes play roles in lipid metabolism.
			Kamada et al., 2002	LDs localized w/in microglia in ischemic core and astrocytes in penumbra.
	Choroid plexus	Astrocyte	Ueno et al., 2001	LD frequently found in several brain regions of senescence-accelerated mice.
Mouse	Frontal lobe	Macrophage	Sturrock, 1983	About half of macrophages in the brains studied were distended due to excess LDs or foamy aggregations.
	Medial temporal	Various	Sturrock, 1988	LDs appeared in the choroid plexus with increased age.
	Cortex	Astrocyte	Nakajima et al., 2019	Inhibition of DAG acyltransferase blocks LD formation and lipotoxic cell death
		Autolysosome	Yang et al., 2014	Lipids impeded macroautophagy and clearance in an AD mouse model.
	Hippo-campus	Neuron, glia	Chali et al., 2019	Neuronal loss and glial cell proliferation associated with changes in lipid related transcripts.
			Ioannou et al., 2019	Neurons expelled fatty and nearby astrocytes engulfed and stored them as LDs.
		Microglia	Marschallinger et al., 2020	LD-accumulating microglia were defective in phagocytosis, produced high levels of ROS, and secreted pro-inflammatory cytokines.
	Hypothalamus	Astrocyte	Kwon et al., 2017	Hypothalamic astrocytes accumulated LDs and had increased cytokines.
		Tanycyte	Rawish et al., 2020	There was high LD signal in mice fed a high at diet, which returned to normal under telmisartan treatment.
			Kim et al., 2020	A high fat diet increased the number and size of LDs.
		Neuron	Crespo et al., 1995	Neurons stimulated with CDP-choline displayed LDs in their cytoplasm.
	Mesencephalon	Neuron	Han et al., 2018	Lipid dysregulation in PD involved upregulated expression of Plin4, increased LD deposition, and loss of neurons.
	Neo-striatum	Various	Sturrock, 1980	Pericytes contained LDs in the neostriatum, indusium griseum, and anterior commissure at various ages.
	SVZ	Neuronal stem	Bouab et al., 2011	Cells with increased numbers of large LDs showed heightened signs of quiescence and metabolic disturbance.
			Hamilton et al., 2015	Impaired FA metabolism suppressed neural stem cell activity
	White matter	Various	Liberski et al., 1989	Macrophages in mice with Creutzfeldt-Jakob disease were filled with LDs.
	Whole brain	Glia	Ogrodnik et al., 2019	Mice fed high fat diets had increased LDs and cells with more LDs were more likely to be senescent.
		Neuron	Hamilton et al., 2010	Postmortem AD brains and 3xTg mice were shown to accumulate neutral lipids in ependymal cells.
		Various	Shimabukuro et al., 2016	Lipid-loaded cells displayed a variety of distinct phenotypes based on their location and numbers increased with age.
	Cortex	Glia	Cabirol-Pol et al., 2018	ND23 knockdown in glial cells created massive LD accumulation and induced brain degeneration.
			Kis et al., 2015	LDs were localized in glia and enriched in the cortex.
Fly	Whole brain	Neuronal stem	Bailey et al., 2015	LDs played an antioxidant role in neural stem cells by reducing ROS and protecting against peroxidation.
	Hypothalamus	Neuron	Nakai et al., 1979	Large LDs were found in CSF contacting neurons.
	Various	HeLa	Papadopoulos et al., 2015	LD targeting may contribute to HSP pathogenesis.
		Glia	Meng et al., 2015	ROS and neuronal mitochondrial dysfunction contributed to LD accumulation prior to neurodegeneration onset.
		Neuron	Calderon-Garciduenas et al., 2002	Air pollution caused ApoE-positive LDs to be deposited in SMC and pericytes
Other	Zebra-fish	Embryo	Arribat et al., 2020	Loss of spastin resulted in a higher number of smaller LDs.
	*In vitro*	Glia	Lucken-Ardjomande Hasler et al., 2014	GRAF1α was found on LDs in primary glial cells that were fed oleic acid. Overexpression of GRAF1a promoted LD clustering and perturbed lipolysis.
*In vitro*	*In vitro*	Glia	Khatchadourian et al., 2012	LPS treated microglia accumulated LDs and Plin2 colocalized with droplets.
			Lee et al., 2017	An increased BBB Ki induced LD formation, activated stress pathways, and increased inflammatory cytokines.
			Farmer et al., 2019	E4 astrocytes have increased lipid content compared to E3
		HeLa	Edwards et al., 2009	Spartin may be recruited to LDs.
			Hooper et al., 2010	Lack of spartin expression contributes to Troyer syndrome.
			Cole et al., 2002	PD mutations in α-syn showed less variable LD distribution and less TG turnover. α-syn formed oligomers within cells and associated with LD.
		N41	Libby et al., 2015	Cells treated with LPL accumulated lipid into droplets.

*These papers have been classified by the model organism that was used, area of the brain that was studied, and cell type of focus.*

## Anatomical Structures Associated With LDS in the Brain

The subventricular zone (SVZ) has surfaced as a key region for LDs in the brain. The SVZ lines the wall of the lateral ventricles and is composed of neural stem cells that are capable of differentiating into various CNS lineages (Doetsch et al., 1999). This highly active and heterogeneous cellular region is an energetically needy zone, and it is reasonable to expect ample energy stores are on board in order to meet its energetic needs (Stoll et al., 2015). It was first shown that large LDs were found in the ependyma of the SVZ (Doetsch et al., 1997). This was accomplished using an electron microscopy approach that detailed the associations of various SVZ cell types with confirmatory immunostaining.

Another group found that the LD lipase ATGL, which is encoded by the patatin-like phospholipase domain-containing protein 2 (PNPLA2) gene, is highly active in the SVZ and choroid plexus. Furthermore, mutating PNPLA2 led to a significant increase in LD formation in both areas (Etschmaier et al., 2011). This study was the first to report a function of PNPLA2 in the brain and describe its regional role in maintaining cerebral lipid metabolism. Additionally, knock out of the GTPase regulator associated with focal adhesion kinase-1 (GRAF1) induced LDs in the brains of post-natal day seven pups (Lucken-Ardjomande Hasler et al., 2014). Apart from studies of LDs in the brain arising from genetic alterations, it was found that LDs accumulate in the SVZ progressively with age; 12-month-old mice showed a nearly two-fold increase in LDs in the SVZ compared to 3-month-old mice (Bouab et al., 2011).

The SVZ has also been shown to harbor LDs in the context of AD. Hamilton and colleagues showed that both an AD mouse model and AD human post-mortem tissue accumulate LDs along the lateral ventricle (Hamilton et al., 2015). Using tandem mass spectrometry, the LD contents were identified, revealing high concentrations of oleic acid-enriched TAGs (Hamilton et al., 2015). Interestingly, direct infusion of oleic acid into the lateral ventricle was sufficient to induce LD formation along the SVZ, but insufficient to impair neurogenesis (Hamilton et al., 2015). Another group found the choroid plexus to have more LDs as AD progressed in human post mortem tissue (Yin et al., 2019). Further studies are needed to clarify the role of LDs in the SVZ in normal aging and neurodegeneration.

Although the SVZ is the most extensively studied brain region with regards to LD formation, other structures such as the frontal cortex, hippocampus, olfactory bulbs, and hypothalamus have been shown to accumulate LDs (Table 1). In fact, prior studies on the hypothalamus indicate that LDs may affect certain processes such as satiety (Kaushik et al., 2011). In this study, Kaushik demonstrates that autophagy during starvation leads to the mobilization of neuronal lipids which can then increase food intake though the upregulation of agouti-regulated peptide. This is just one of many studies which explore the wide variety of brain regions and biological processes which LDs can affect (Table 1).

## What Causes Lipid Droplets to Form in the Brain?

### Aging

Lipid droplets appear to accumulate in the brain during the normal process of aging. For example, analysis of microglia from 20-month-old mice revealed an abundance of BODIPY+ cells in comparison to a matched 3-month-old cohort (Marschallinger et al., 2020). Analyses of human tissue (postmortem) also revealed that PLIN2+ Iba1+ microglia were more frequent in an aged (67-years-old) individual than in a young (22-years-old) individual (Marschallinger et al., 2020). A significant increase in LDs has also been observed in the pia mater, cortex, and striatum in 18-month-old mice as compared to middle aged mice (Shimabukuro et al., 2016). Furthermore, an electron microscopy analysis of the basement membrane of the blood brain barrier (BBB) in 6-month-old versus 24-month-old mice showed an age-dependent accumulation of LDs which caused significant thickening of the basement membrane (Ceafalan et al., 2019). On the contrary, LDs are more commonly found in perivascular cells in middle age and then seem to shift toward the parenchyma in old age (Shimabukuro et al., 2016). Given these findings, age appears to regulate LD accumulation and regional deposition.

### Inflammation

From *in vitro* studies of LDs to *ex vivo* brain imaging, inflammation has repeatedly been associated with LD formation as both a cause and as an effect (Bozza and Viola, 2010). Lipopolysaccharide (LPS), a commonly employed pro-inflammatory stimulus, has been shown to increase the number and size of LDs in microglia (Khatchadourian et al., 2012). PLIN2 was shown to colocalize with these droplets, providing more evidence that PLIN2 is an LD-associated protein that can be considered a marker for both LDs and inflammation in the brain. This was repeated recently in microglia-derived BV2 cells and expanded into an *in vivo* model of LPS treatment. That study by Marschallinger and colleagues found that more microglia contained LDs in LPS-treated mice when compared to non-treated controls (Marschallinger et al., 2020). To assess how the vasculature might affect LDs in the brain, Lee and colleagues *i.v.* infused triglyceride-rich lipoproteins (TGRL) and lipoprotein lipase into mice and found increased BBB permeability, therefore indicating that hyperlipidemia may increase lipid spill-over into the brain. To test how this treatment affected resident brain cells, they treated normal human astrocytes with the TGRL lipolysis products and found increased LD formation (Lee et al., 2017). Another study found that palmitate treatment of isolated primary astrocytes increased inflammatory markers including TNF-alpha, IL-1 beta, IL-6, and MCP-1 in addition to Oil Red O (a fat-soluble dye) staining and PLIN1 and PLIN2 transcription. Interestingly, treatment of microglia with conditioned media from lipid-loaded astrocytes enhanced microglial chemotaxis through a CCR2-MCP1 mechanism (Kwon et al., 2017). These data suggest that LD-associated astrocyte inflammation may subsequently signal to microglia to augment the inflammatory response. However, it remains to be seen whether inflammation causes LDs, LDs cause inflammation, or both.

### Oxidative Stress

Intracellular reactive oxygen species (ROS), as well as ectopic treatment with oxidative stressors such as hydrogen peroxide, induce LD formation in various cell types in the periphery (Lee et al., 2013, 2015; Jin et al., 2018). Similarly, increased oxidative stress in the brain appears to drive LD accumulation in a cell-specific manner. For example, neuronal hyperactivity from trauma or chemogenetic activation increases glial LD accumulation (Ioannou et al., 2019). These LD-laden glia upregulate genes to neutralize the peroxidated lipids generated by activated neurons. Astrocytes in particular appear to be uniquely suited for ROS management due at least in part to fatty acid binding protein 7 expression (Islam et al., 2019). Liu and colleagues first proposed this neuron-astrocyte metabolic coupling model in which neurons under stress export oxidized lipids to astrocytes as a means of neuroprotection (Meng et al., 2015; Liu et al., 2017). LDs in glia may then be viewed as indirect indicators of neuronal damage from oxidative stress. Protective LD formation in glia has also been observed in the SVZ niche, where glia protect neuroblasts from peroxidation and thereby promote neural stem cell proliferation (Bailey et al., 2015). Therefore, oxidative stress appears to be a driver of LD formation in the brain both under formative physiological processes during neuronal development, as well as in diseases associated with increased neuronal oxidative stress.

## What Neurodegenerative Disorders Have Been Linked to Lipid Droplets?

### Amyotrophic Lateral Sclerosis

Amyotrophic lateral sclerosis (ALS) is the most common form of motor neuron disease (Tiryaki and Horak, 2014). ALS is characterized by the progressive degradation of motor neurons in the CNS, leading to the inability to both initiate and control muscle movement. In addition to genetic mutations, altered metabolic function in ALS has been observed in cellular processes implicated in ALS pathology such as cell stress and energy homeostasis (Vandoorne et al., 2018).

Recent studies interested in lipid metabolism have shed light on connections between LDs and ALS pathology (Pennetta and Welte, 2018). For example, it is known that mutations in the human VAMP-associated protein B (hVAPB) cause ALS, although the disease-causing mechanism itself remains unclear (Sanhueza et al., 2015). Sanhueza and colleagues performed a genome-wide screen in *Drosophila* to identify pathways involved in hVAPB-induced neurotoxicity and found that the list of modifiers was mostly enriched for proteins linked to LD dynamics. One modifier highlighted in this study was acyl-CoA synthetase long-chain (Acsl). Acsl promotes LD biogenesis and its downregulation reduces LD nucleation which decreases the size and number of mature LDs (Kassan et al., 2013). Furthermore, another group showed that gain of function mutations in the LD protein seipin contributed to motor neuron disease symptoms in mice (Yagi et al., 2011; Sanhueza et al., 2015). Together these studies suggest that impaired LD biogenesis may be an important pathological aspect to hVAPB-mediated ALS.

A mutation that contributes to an early onset form of ALS occurs in the gene SPG11 and affects lysosome recycling (Branchu et al., 2017). Branchu and colleagues observed intracellular lipid accumulation followed by lipid clearance from lysosomes into droplets in wild-type (WT) mice. However, in Spg11 knockout mice, there was a significantly slower rate of lipid clearance and a decrease in LD size and number. Another study that implicates aberrant lysosomal function as a contributor to ALS reported that the C9orf72 gene plays a key role in metabolic flexibility in times of stress/starvation (Liu et al., 2018). They found that loss of C9orf72 led to an increase in LDs, and that starvation-induced changes in lipid metabolism were mediated by coactivator-associated arginine methyltransferase (CARM-1). Since CARM-1 regulates lysosomal function and lipid metabolism, these results suggest that the dysregulation of lipid metabolism, including the aberrant accumulation of LDs, could contribute to ALS pathology.

Similar to studies linking LDs and peroxidation to AD, there also appears to be a connection between LDs and cellular stress in ALS. Bailey et al. showed in *Drosophila* that ROS accumulation increased glial LD content, and that when glia were unable to produce LDs, neuroblasts experienced peroxidative damage (Bailey et al., 2015). Additional work by Simpson et al. provided a link between LDs, peroxidation, and ALS by showing a positive correlation between lipid peroxidation markers in ALS patient cerebrospinal fluid and disease burden (Simpson et al., 2004). Thus, LD dynamics may contribute to ALS, potentially through a mechanism in which glia are unable to protect neurons through normal lipid accumulation and storage mechanisms (Pennetta and Welte, 2018).

### Huntington’s Disease

Huntington’s disease (HD) is a hereditary neurodegenerative disease caused by a mutation in the huntingtin gene (Htt) that then codes for the Htt protein (McColgan and Tabrizi, 2018). Mutant Htt contains an expanse of repeating glutamines at the N terminus, thus causing Htt oligomerization and aggregation that leads to neuronal death. A wide range of metabolic abnormalities characterizes HD, including alterations in autophagy (Croce and Yamamoto, 2019). Martinez-Vicente et al. (2010) found that macroautophagy is compromised in cellular and mouse HD models and in HD patient-derived tissues, as evidenced by the inability to recognize and properly sequester unneeded cellular components. Autophagosomes in HD models failed to recognize and load excess cargo, thus causing autophagic cytosolic components to have a slower turnover leading to toxic accumulation of lipid in cells. They also observed an increase in LD number and area in fibroblasts, hepatocytes, striatal cells, and primary neurons from a HD mouse model (Qhtt mice). Furthermore, striatal tissue from advanced-stage HD patients had increased Oil Red O staining density when compared to age-matched controls (Martinez-Vicente et al., 2010). The authors hypothesized that increased LD content in HD cells could be due to their reduced ability to recognize and degrade excess lipid by macroautophagy.

Aditi et al. (2016) used transgenic *Drosophila* expressing the mutated form of human Htt in neurons to better understand HD energetics. They found that when compared to controls, diseased flies exhibited a characteristic pattern of weight change that is correlated with HD progression, as evidenced by altered lipid concentrations over time. Interestingly, and in contrast to the above study by Martinez-Vicente’s group, flies had high levels of lipid at disease onset and low levels at the terminal stage in neurons. This finding was also true for abdominal body fat cells, despite only expressing mutant Htt in neurons. LD size mirrored the overall lipid levels, with large LDs being found in 3-7-day-old diseased adults and small LDs forming by days 11-13 in diseased flies. These findings suggest that mutant Htt leads to dysregulated lipid metabolism in addition to neurodegeneration. However, further studies in both mammalian and fly models are needed to fully elucidate the role LDs may play in the onset of HD.

### Parkinson’s Disease

Parkinson’s disease is caused by a loss of dopaminergic neurons and leads to abnormal brain activity and symptoms such as tremors, bradykinesia, and limb rigidity (Kalia and Lang, 2015). Lewy bodies are pathological hallmarks of PD that are found in pre-synaptic terminals of neurons. These Lewy bodies contain aggregates of the α-synuclein protein which has been shown to accumulate on LD phospholipid surfaces, slowing lipolysis of LDs (Cole et al., 2002). Cole and colleagues also found that two mutant forms of α-synuclein, A30P and A53T, showed decreased capacity to reduce LD turnover in neurons compared to WT α-synuclein. These results suggest LD turnover in neurons is contingent on proper α-synuclein function, and that LD lipolysis may contribute to PD (Cole et al., 2002). However, these findings are complicated by the work of Outeiro and Lindquist (2003), who found that both WT and A53T α-synuclein caused an accumulation of LDs in yeast, whereas A30P synuclein did not. These findings suggest that LDs may play a cell-specific role in PD pathology and call for further study in this area.

Integrated genome wide association studies have found that key mechanisms of PD pathogenesis (oxidative stress response, lysosomal function, ER stress response, and immune response) rely heavily on genes regulating lipid and lipoprotein signaling. For example, Klemann and colleagues found that lipid and lipoprotein signaling is regulated by the same processes involved in dopaminergic neuron death, and found deficient signaling to be associated with increased risk for PD (Klemann et al., 2017). Scherzer et al. found that genes related to lipid metabolism and vesicle-mediated transport had the largest effects on increasing α-synuclein toxicity in yeast (Scherzer and Feany, 2004). This same group also classified a variety of genes related to α-synuclein expression in *Drosophila*, and again found that lipid-related genes were strongly associated with this process. The authors proposed that dysregulation in lipid processing may be an indicating factor of problems caused by A30P α-synuclein toxicity (Scherzer et al., 2003).

Additional studies have begun to investigate lipid dyshomeostasis in PD more deeply. For example, suppression of the oleic acid generating enzyme stearoyl-CoA-desaturase (SCD) was recently found to be protective against α-synuclein yeast toxicity, and SCD knockout models in roundworms was shown to prevent dopaminergic neuron degeneration (Fanning et al., 2019). Specific genes related to lipid regulation have also been identified. ATPase cation transporting protein 13A2 (ATP13A2) functions in cation transport within the cell. Mutations to ATP13A2 are associated with PD and overexpression of ATP13A2 showed a decrease in various forms of lipids *in vitro* (Marcos et al., 2019). When looking at these findings together, it is evident that LDs and lipid homeostasis play a more significant role in PD than originally thought. This line of thought is supported by Fanning et al. (2020) who stated that α-synuclein toxicity and cell trafficking defects have been associated with aberrations in LD content and distribution. PD has classically been believed to be a “proteinopathy,” but with many of the recent discoveries, lipid dyshomeostasis is rapidly becoming one of the fundamental characteristics of this disease (Fanning et al., 2020).

### Alzheimer’s Disease

Alzheimer’s disease is the most common form of dementia worldwide (Karantzoulis and Galvin, 2011). When Alois Alzheimer wrote his seminal paper in 1907 describing the case of Auguste Deter, he noted three neuropathological hallmarks. He found “striking changes of the neurofibrils” and “minute milliary foci caused by deposition of a particular substance in the cortex.” He also observed glial changes and stated, “many glia include adipose inclusions” (Alzheimer, 1907; Alzheimer et al., 1995). While the first two findings have been studied extensively by scientists interested in the contribution of tau and amyloid to disease progression, the finding of glial lipid accumulation has largely been overlooked. It wasn’t until recently that this phenomenon of increased lipid accumulation in AD was revisited and examined. Hamilton et al. (2015) helped renew interest in this phenomenon in a report describing increased LD formation in the SVZ of both 3xTgAD mice and human AD samples which correlated with defects in neurogenesis. Interestingly, acute administration of intracerebral oleic acid mimicked the LD phenotype of the 3xTgAD mice, but did not alter SVZ neuron viability, suggesting that disease-associated LD accumulation is not simply a result of environmental lipid exposure. Derk et al. (2018) found a highly significant increase in both neutral lipid and diaphanous 1 (DIAPH1) expression in myeloid cells in AD brains. DIAPH1 mediates signaling for the receptor for advanced glycation end products (RAGE), an inflammatory ROS-producing pathway. This apparent correlation between neutral lipid accumulation and inflammatory signaling suggests that LDs may be key players in cerebral inflammatory responses. A recent study further validated this model, where it was discovered that astrocytes uniquely upregulate ROS management genes, seemingly to manage the import of neuron-derived lipid oxidation products (Ioannou et al., 2019). This study showed that neuronal hyperactivity alone was sufficient to initiate neuronal lipid peroxidation, neuronal lipoprotein export, and subsequent management and storage of peroxidized lipid as LDs in astrocytes. Further work done by van der Kant et al. (2019) showed that CE, which can be incorporated into LD cores through normal LD biogenesis, increase the accumulation of phosphorylated tau (p-tau) by reducing proteasome activity. The study showed that both statins and an allosteric activator of cholesterol 24-hydroxylase (efavirenz) helped lower p-tau levels in human neurons by reducing CE concentrations, thereby providing a potential mechanistic link between LDs and AD neuropathology.

A role for apolipoproteins as shuttles for oxidative waste from neurons has been described in the brain (Liu et al., 2017). Additionally, different isoforms of Apolipoprotein E (ApoE) were shown to have altered efficiency for lipid shuttling. ApoE4-laden lipoproteins appeared to be less efficacious at the delivery of lipotoxic products to glia than lipoproteins associated with ApoE3.

This is particularly interesting in light of the E4 allele of *APOE* being the strongest genetic risk factor for the development of late onset AD. Our group recently showed that astrocytes expressing E4 preferentially accumulate and utilize LDs for energetic needs (Farmer et al., 2019). Additionally, transcriptional profiling of glia derived from human iPSC lines harboring homozygous E4 or E3 alleles showed that the majority of differentially expressed genes in astrocyte-like cells involved lipid metabolism and transport (Lin et al., 2018). This group also found that a phenotype of E4 astrocytes included the accumulation of intracellular and extracellular cholesterol. They hypothesized that since cholesterol is responsible for a wide range of functions in the brain, altered cholesterol metabolism in E4 glia may be associated with pathological phenotypes in neurodegenerative disorders. Conditioned media from E4 astrocytes has been shown to induce LDs in other cell types, suggesting that E4 may also act extracellularly to induce LD formation (Tambini et al., 2016). Finally, neutral lipid staining of the choroid plexus in post-mortem AD brains proposed LDs as central hubs of an ApoE-mediated complement-cascade regulation (Yin et al., 2019). The authors found ApoE to bind to complement component 1q (C1q), a protein complex that binds antigen-antibody complexes, on LDs in the choroid plexus. Since C1q protein is involved in the activation of the classical complement pathway, this interaction effectively keeps the complement system of the immune system in check at the CNS/vasculature interface. While all ApoE isoforms showed equal binding affinity for C1q, post-mortem mice and human E4 brains were shown to accumulate LDs more abundantly. Interestingly, these lipid deposits significantly correlated with neuropathological staging of AD, pointing to *APOE* regulation of the complement cascade at the choroid plexus niche as a novel hypothesis for AD pathogenesis.

### Hereditary Spastic Paraplegia

Hereditary spastic paraplegia (HSP) is a group of inherited neurological disorders that cause muscle weakness and tightness, primarily in the legs, through the degeneration of long corticospinal axons (Lo Giudice et al., 2014; de Souza et al., 2017). Studies of HSP-causing proteins suggest a link between lipid metabolism and the development of disease. For example, DDH2 domain containing protein 2 (DDHD2) is a triglyceride hydrolase in the brain that is implicated in recessive complex HSP. The systemic genetic knockout and pharmacological inhibition of DDH2 resulted in large-scale accumulation of LDs within the CNS, but not elsewhere (Inloes et al., 2018). These data indicate a link to TAG metabolism, as the inhibition of DDHD2 affects lipid homeostasis and LD number.

One of the most common genetic mutations involved in HSP occurs in the microtubule severing protein spastin (Papadopoulos et al., 2015; Branchu et al., 2017). The M1 isoform of spastin contains a LD targeting sequence which contributes to protein targeting to LDs and LD sorting at the ER. Additionally, spastin deficiency in *Drosophila* and *C. elegans* altered LD number and TAG content. This study suggests that LD processing may contribute to the pathogenesis of HSP (Papadopoulos et al., 2015). Additionally, a study by Arrabit et al. further identifies spastin as a regulator of LD dispersion and dynamics. This group showed that mutations in the spastin M1 isoform induced ER reorganization in HeLa cells. This reorganization subsequently disrupted spastin’s ability to disperse LDs throughout the cell and aberrantly modulated neutral lipids and phospholipids on membranes throughout the muscle and brain (Arribat et al., 2020). Furthermore, embryonic zebrafish cells that were treated with oleic acid (a common method to induce LD formation) indicated that a loss of spastin resulted in a higher number of smaller LDs, therefore suggesting differential generation, and/or dispersion of LDs (Arribat et al., 2020). In addition to identifying potential new HSP biomarkers, this study also proposes that HSP-causing mutations impacts lipid profiles and LD networks.

Several groups have also forged a connection between the protein spartin and LD regulation. Spartin is a multi-functional unit that associates with LD, and a lack of spartin expression contributes to a HSP form called Troyer syndrome (Hooper et al., 2010). In one study by Edwards et al. (2009) spartin surrounded LD clusters in oleic acid treated HeLa cells, thus suggesting that spartin is recruited to LDs. Additionally, in spartin knockout mice, female mice had increased LDs number and higher perilipin protein levels in adipose tissue (Renvoisé et al., 2012). Hooper and colleagues further demonstrated an interaction between spartin and LDs. Their experiments in HeLa cells showed that spartin binds to and recruits the ubiquitin ligase atrophin-1-interacting protein 4 (AIP4) to LD. This interaction subsequently promotes the ubiquitination of PLIN2. Since PLIN2 resides on LD membranes and regulates TAG turnover, spartin may play a role in LD regulation in cells and contribute to Troyer syndrome pathology (Hooper et al., 2010). Furthermore, another group reported binding interactions between spartin and protein kinase C interacting proteins (ZIPs) at the surface of LDs. Spartin-expressing HEK-293 cells exhibit co-localization of spartin with ZIP1 and ZIP3 on LDs as shown by superimposition of spartin GFP-tagged fluorescence and Oil Red O fluorescence (Urbanczyk and Enz, 2011). Interestingly, in the absence of spartin, no ZIP proteins were detected on the LD surface (Urbanczyk and Enz, 2011). These collective findings suggest that impaired LD metabolism might be one mechanism that contributes to Troyer Syndrome.

## Regulation of LDS in the Brain

Lipid droplets in the brain may be regulated differently than more traditional LD niches. DDHD2 action in HSP implicates this protein as an important player in CNS lipid metabolism but also shows a dichotomy between peripheral LDs and central LDs. The BBB forms a tight and regulated gate for lipid import and export into the brain, and thus there are separate pools of lipids that constitute the intracellular LDs located in cells in the CNS versus the periphery. Lipid contents of the cell types that make up the BBB appear to regulate its permeability (Andreone et al., 2017; Ceafalan et al., 2019). Since enzyme mutations of key lipases are manifested differently in the brain and given the existence of lipid-regulatory mechanisms between the periphery and the CNS, it is possible that the two pools are controlled by separate mechanisms. Investigators should be cautious when applying canonical peripheral LD pathways to brain LD biology until more evidence emerges for conserved physiological pathways between the brain and the periphery.

Sex-specific regulation of lipid metabolism and LDs in the brain should also be considered. In one study, neurons of male rats were shown to be more vulnerable to starvation than neurons of female rats, with male neurons exhibiting decreased mitochondrial respiration, increased autophagosome formation, and increased cell death when compared to females (Du et al., 2009). On the other hand, the same study showed that female tolerance of starvation conditions was associated with increases in fatty acid content and LD formation in order to prolong cell survival. These findings suggest that central LD dynamics may be sex dependent. Potential sex differences should be considered in future studies in order to better understand the contribution of sexually dimorphic features to cerebral LD metabolism and its relevance to disease susceptibility and pathology.

## Future Directions and Concluding Remarks

Several important knowledge gaps remain in our understanding of the role of LD biology in the pathophysiology in neurodegenerative disease. One important future direction for the field is to better understand the full composition of lipids comprising brain LDs – in both healthy and diseased states. A clearer picture would likely be revealed by the precise distribution of lipid classes in distinct brain regions, as well as in specific neurodegenerative diseases. However, their relatively small size and the diversity of LDs by age, cell type, and disease type complicate the compilation of a lipid profile of LDs. Additionally, *in vivo* analysis of lipids in general is challenging, as they are a diverse class of macromolecules and are relatively insoluble (Cornett et al., 2007). However, improved techniques in mass spectrometry provide new ways to document the lipid composition in LDs. For example, matrix-assisted laser desorption/ionization imaging mass spectrometry (MALDI-IMS) allows for anatomically-specific direct detection of lipids within membranes through the production of lipid-derived ions (Fuchs et al., 2010). Since MALDI can detect phospholipids, sphingolipids, and glycerolipids, this technique provides the opportunity for an in depth and spatially-resolved profile of LDs (Murphy et al., 2009). The continued use of powerful imaging techniques such as MALDI will be important to providing a better understanding of lipid class distribution in cerebral LDs.

Lipid droplets are cellular fuel stores, markers of inflammation, signaling hubs, protective waste reservoirs for hyperactive neurons, products of lysosomal dysregulation, and hallmarks of age. With such a wide array of roles, it is not surprising that LD accumulation has been linked to trauma, neurodegeneration, and aberrant cerebral metabolism. LDs are promising targets for novel investigations of neurological disease diagnosis and therapeutics. Therapeutic treatments could be targeted at restoring lipid balance, decreasing droplet levels, or improving other aspects of lipid metabolic pathways. Further study on LDs and lipid metabolism will be essential in advancing our knowledge of cerebral metabolism, as well as the multifaceted etiologies of neurological disease.

## Author Contributions

BF initiated this work and drafted the manuscript. BF, AW, JK, and LJ wrote the manuscript. AW and JK performed the literature search for Table 1. All authors read and approved the manuscript in final form.

## Conflict of Interest

The authors declare that the research was conducted in the absence of any commercial or financial relationships that could be construed as a potential conflict of interest.
